# Proximal avulsion of the hamstring in young athlete patients: a case series and review of literature

**DOI:** 10.1007/s00590-024-04096-1

**Published:** 2024-10-16

**Authors:** Alberto Castelli, Matteo Parenti, Gianluca Tirone, Marco Spera, Flavio Azzola, Giacomo Zanon, Federico Alberto Grassi, Eugenio Jannelli

**Affiliations:** 1 Department of Clinical, Surgical, Diagnostic and Pediatric Sciences, University of Pavia, 27100 Pavia, Italy; 2 Present Address: Orthopedic and Traumatology Clinic, IRCCS Policlinico San Matteo Foundation, 27100 Pavia, Italy; 3 Università degli Studi Di Parma, 43121 Parma, Italy; 4 Istituto Clinico Sant’Anna, 25127 Brescia, Italy

**Keywords:** Hamstring Injuries, Proximal Hamstring Avulsion, Functional outcome, Sport, Return to sport, Athletes

## Abstract

Hamstring injuries are a frequent occurrence of athletes, leading to a stop in practice and long-term alterations in sports performance. About 12% of these lesions involve avulsion at the level of the proximal insertion that can be complete (about 6%) or partial. Starting from an epidemiological and treatment evaluation of these lesions in literature, the aim of this study was to examine the functional outcomes and the rate of "return to play" in a population composed of athletes of various levels who have undergone surgery to reinsert the hamstring muscles at the ischial insertion, for a complete detachment of one or more tendon heads. Therefore, a retrospective study was carried out where 18 patients treated at the Orthopedics and Traumatology Unit of the I.R.C.C.S. San Matteo in Pavia (Italy) were identified in a time span ranging from March 2012 to August 2020. The sample was analysed taking into account age, sex and risk factors, as well as the pathophysiology and anatomy of the injury using the Wood classification, the time elapsed before surgery, the duration of the rehabilitation protocol and the possible return to sports activity, comparing the level of sports performance in the pre- and post-operative period using the Tegner Activity Score (TAS). Different post-operative outcome evaluation scores (Perth Hamstring Assessment Tool PHAT and Lower Extremity Functional Scale LEFS) were also compared with each other in order to find a correlation with the real level of return to sporting activity. The mean age at surgery was 26.4 11.6 years. The population is composed of 14 males (77.8%) and 4 females (22.2%). All 18 patients returned to sports following surgery (100%). Of these patients, 17 (94%) maintained a level of sports performance equal to that before the injury. 100% of patients rated the outcome of the surgery as satisfactory. This study has shown that Hamstring reinsertion surgery is a correct indication in all athletes, allowing them a satisfactory return to sports practice.

## Introduction

### Background

Hamstring-related lesions account for 12% to 29% of all injuries among athletes [1]. Most injuries involve muscle or myotendinous junction strains, which are often treated conservatively. Avulsion of the hamstring tendons from their proximal origin at the level of the ischial tuberosity (Proximal Hamstring Avulsion, PHA) is a less common but more severe scenario. It has been estimated that PHAs make up about 12% of all hamstring injuries, however only 8% of these consist in a full lesion. With prolonged pain, decreased functionality, neurological symptoms from sciatic nerve involvement, and an inability to engage in sports, conservative therapy of PHA often have poor clinical outcomes. For this reason, the majority of authors suggest surgical repair in cases of complete PHA [2].

Most hamstring injuries sustained during athletic competition result from non-contact movements like running and sprinting [3]. These activities can result in acute tendon avulsion injuries or lacerations of biomechanically sensitive areas, such as the intratendinous segment or myotendinous junction, due to quick, explosive motions combining hip flexion and ipsilateral knee extension. The long head of the biceps femoris is frequently the injured muscle in these kinds of events.

Less frequently, hamstring injuries are brought on by chronic degenerative tears resulting from recurrent microtrauma and/or excessive straining of the tendon units, together with inadequate repair inside the tendon part. Dancers and gymnasts who simultaneously overstretch their hamstrings while performing excessive hip flexion and knee extension typically suffer from this sort of injury, as do endurance athletes who often suffer from insertional tendinopathy. These often affect the free proximal semimembranosus tendon, in contrast to acute injuries. Patients in these circumstances frequently report less intense pain, worsening hamstring weakness, and a gradual loss of function.

These injuries are regarded as career-threatening in athletes with high functional demands and can result in early retirement. Since older persons continue to be physically active and engage in recreational sports activities, hamstring injuries are not just a problem for professional athletes but affect also the general population.

Clinical awareness of these lesions can be improved by having a thorough understanding of epidemiological data; however, the literature lacks comprehensive data on patient demographics, damage mechanisms, and injury patterns.

Our study aims at filling a gap by reviewing recent literature regarding similar lesions and treatments.

The main goal of the study was the evaluation of the return to sporting activity following surgical treatment of a proximal avulsion of the hamstring muscles.

Secondary goal included verifying the functional recovery in the post-operative period, submitting two functional scores (PHAT and LEFS) to each patient and understanding the relationship between those and other parameters such as age, risk factors, duration of physiotherapy protocols and mechanism of injury.

### Risk factors

A recent study published in the BMJ investigated 179 potential risk factors in a dataset of 8,319 hamstring injuries (of which 967 were recurrences), in over 71,000 athletes [4].

Modifiable risk factors and non-modifiable risk factors were used to categorize the risk variables under investigation.

The meta-analysis revealed that advanced age and any prior hamstring injury, including strains, muscle rips, and avulsions, were the primary risk factors. Athletes who have experienced a hamstring injury in the past are 2.7 times more likely to experience an injury once again, and their risk increases to about 5 times if the prior injury happened during the same season [5]. This happens because during recovery, the development of scar tissue reduces the tendon’s flexibility, making it harder for it to contract and raising the likelihood of a subsequent injury in the area of scar tissue formation [6–8].

Injuries caused by a single sudden trauma, as opposed to smaller cumulative injuries, tend to be more severe and mostly affect younger people while sprinting and older individuals while engaging in in high-risk sports like water skiing. Long-distance running can predispose to chronic tendinopathy, which is likely the result of several microscopic events that damage the tendons, putting a person at risk for injury [9].

Non-modifiable factors include advanced age (> 25 years). Due to increased exposure over time, age may have an effect on the likelihood of hamstring injuries in athletes. As athletes get older, they are subjected to higher mechanical stresses and are therefore more likely to experience injury-causing processes. Older athletes may be more vulnerable to injury due to structural (such as altered fibre architecture, fibre type, cross-sectional area, and stiffness [10]) and neurological (such as high-threshold denervation of the motor units) changes brought on by ageing.

Although the cause of this is unknown, the risk rises annually after the age of 25, Modifiable risk factors include physical strength asymmetry (taking into account both the difference between ipsilateral and contralateral as well as the strength ratio between the hamstrings and quadriceps), flexibility and exhaustion; with high-speed running and football having the highest risk levels.

The risk of recurrent injury may also be worsened by weakness deficits, decreased hamstring flexibility, and palpable discomfort after returning to sporting activities. Specific MRI indicators were unable to accurately predict the risk of recurrence [4, 5].

### Physiopathology

From a pathophysiological point of view hamstring injuries are classified into two forms based on their mechanism of injury: type 1 or “high-speed running type” and type 2 or “stretching type”. Type 1 injuries happen when you run at or near your maximum capacity. Type 2 injuries arise as a result of movements involving severe hip flexion and ipsilateral knee extension [11].

## Materials and methods

This retrospective study has been conducted by consulting the surgical registries and the outpatients reports of patients subjected to hamstring muscle tendon reinsertion in the Orthopedics and Traumatology Unit at I.R.C.C.S. Policlinico San Matteo of Pavia. The timeframe goes from March 2012 to August 2020.

### Inclusion and exclusion criteria

Patients younger than 40 years old who practised sports at a high competitive level and with a diagnosis confirmed with MRI, who underwent surgical treatment were included in the study.

Patients treated conservatively or with lesion recurrence, older than 40 years, with more than three comorbidities and who had a history of connective tissue disorders were excluded.

The study’s sample includes 18 consecutive patients, all of them being competitive athletes who underwent hamstring tendon reinsertion after partial or total detachment of one or more tendon heads from the ischial insertion.

### Classification

Injury classification was performed using the Wood Classification, which offers the most comprehensive anatomical information and a clinically effective categorization system for proximal hamstring injuries requiring surgical repair [12].

The BAMIC classification system was also used, which offers the most reproducible anatomical approach for stratifying injury severity for any muscle. This system been already employed in various studies to classify and grade hamstring injuries in other studies, using MRI-based methods and exhibiting a high level of inter- and intraobserver agreement [13, 14], stratifying the injury based on its severity. The main limitation is that it doesn’t classify the level of tendon avulsion and does not account for any concurrent neurological conditions [15].

### Surgical technique

All surgeries were performed under general anesthesia in a carefully padded prone position. The ischial tuberosity and the gluteal fold were identified and a transverse 8 cm skin mark was performed. Using these marks, a longitudinal skin incision was made. The posterior thigh fascia was cut through and the posterior lateral cutaneous femoral nerve is identified in order to diminish post-operative superficial numbness. Blunt dissection of the intermuscular plane medial to the biceps femoris was performed to locate the injured intramuscular tendon. The sciatic nerve was always identified, usually deep to the tendon. The proximal and distal ends were identified and approximated with traction sutures, later on 3 to 5 metallic anchors were positioned on the ischial imprint (one anchor per tendon). Once the anchors are secured, a free needle is used to pass the sutures sequentially through the tendon using full thickness stitches.

### Rehabilitation protocol

Post-operative rehabilitation included:

1. Intense physiotherapy with focus on early activation of the posterior thigh muscles: with initial active assisted movement, then against gravity and then with progressive resistance (1–6 months).
2. Protection of the surgical repair until adequate healing (6–8 weeks). Immediately after surgery, the patient used an articulated knee brace, with progressive release of extension until the fourth week.
3. Prevention of excessive stretching and tensioning of the surgical repair, therefore hip flexion with extended knee and flexed torso should be avoided.

After an initial period of abstention from full weight-bearing, the patient will be able to resume gradual weight-bearing with aids compatible with pain.

The return to sport takes place approximately after 16 weeks**.**

### Radiologic outcome evaluation

MRIs were performed pre-operatively in order to diagnose the lesion and at 3 months of follow-up.

All of them were reviewed by an experienced musculoskeletal radiologist. MRI sequences were reviewed and injuries classified according to Wood Classification.

### Clinical outcome evaluation

Follow-up visits were performed for all patients at 7 days, 14 days, 1 month, 3 months, 6 months and 1 year postoperatively.

The sample was examined in terms of age, gender, and risk factors, as well as the pathophysiology and anatomy of the injury, as classified by Wood’s classification, the time elapsed before surgery, and the duration of the rehabilitation protocol. Individual subjects were given a questionnaire that included questions such as previous risk factors, activity and pathophysiological mechanism by which the injury occurred, sport practised, information about the duration and type of rehabilitation protocol used, return to sports activity, and overall patient satisfaction with the surgical intervention. The patients were contacted via phone and additionally presented with two different "self-reported" scores to assess their post-surgical functional recovery.

The Perth Hamstring Assessment Tool (PHAT) was used to assess the clinical outcome of proximal hamstring avulsions treated surgically. The PHAT has a maximum score of 100; a higher score corresponds with greater functionality. The questionnaire rates pain during three different activities (sitting, stretching, and resting) using a visual analogue scale ranging from 0 to 10. The PHAT also employs a five-point scale to determine the maximum amount of time a patient can spend sitting in a chair, driving a car, or running without discomfort; a four-point scale to describe the patient’s current level of activity; and a three-point scale to describe the possibility of discomfort and/or pain in the posterior area of the thigh and/or the lower part of the buttock.

A recent study published in the American Journal of Sports Medicine [16] demonstrated a correlation between PHAT and another more generic score, the LEFS (Lower Extremity Functional Scale), illustrated below. The LEFS is a more general score used to assess functional status in the presence of lower limb musculoskeletal issues. The LEFS is made up of 20 items, with scores ranging from 0 (extreme difficulty/impossibility to carry out activities) to 4 (no difficulty). The final score is obtained by adding the scores of the individual items. The highest possible score is 80, which denotes the absence of all functional limitations [17].

Regarding eventual return to sport activity, the Tegner Activity Score (TAS) was used to compare the sports performance levels in the pre- and post-operative periods. The TAS aims to provide a standardised method for determining activity level prior to and following injury, which can be documented numerically on a scale.

All data regarding the duration of rehabilitation protocols and the weekly number of physiotherapy sessions was gathered in order to assess any potential correlation with the timing of return to sporting activity and functional recovery measured by means of PHAT and LEFS scores.

Furthermore, risk factor of age > 25 years old was established: a possible relationship between this, the duration of the rehabilitation protocol, the return to sporting activity and the functional scores was investigated.

A relationship was between the of mechanism of injury, the duration of the rehabilitation protocol, the return to sporting activity and the functional scores was also investigated.

### Statistical analysis

All statistical analysis were performed on SPSS Statistics version 23.0 (IBM Corp. Released 2015. IBM SPSS Statistics for Windows, Version 23.0. Armonk, NY: IBM Corp.).

Continuous and discrete variables are presented as medians and interquartile range (IQR). Categorical variables are reported as frequencies and percentages.

Regarding the comparison between the two rehabilitation protocol categories, given the small size of the sample it was impossible to resort to distributional hypotheses of normality. Therefore, comparisons between the two groups were carried out using the Mann–Whitney test; to identify the strength and direction of a possible relationship between the functional scores (PHAT and LEFS), Spearman’s rank correlation index was used. The level of statistical significance of all tests, which were conducted two-sided, was set at 0.05. Statistical significance is therefore found for p-values ​​lower than 0.05 (Table 1).

**Table 1:** Patients demographic data

N = 18	
Age at time of surgery	23,5 (18.0–29 0)*
Males n (%)	14(77.8%)
Right side injury n (%)	10 (55.6%)
Sporting injury n (%)	16 (88.9%)
At least one risk factor (age included) n (%)	13 (72.2%)
Surgery at< 6 weeks n (%)	16 (88.9%)
Injury classified by Wood type	
1	2(11.11%)
2	1 (5.56%)
3	1 (5.56%)
4	1(5.56%)
5	11(61.11%)
6	2(11.11%)

*Data expressed as median (IQR) on (%)

## Results

The sample population consists of 14 male patients (77.78%) and 4 female patients (22.22%), accounting for a male:female ratio of 3.5:1. At the time of surgery, the median age was 23.5 years (IQR 18.0–29.0). Eight (44.4%) of the 18 patients were ≥ 25 years old at the time of the injury. In terms of the affected side, 10 individuals (55.6%) were diagnosed with a lesion affecting the right limb (Table 2).

**Table 2:** Distribution of sports disciplines within the sample

Sport	Frequency	Percentage (%)
Soccer	6	33.33
Track and field	6	16.67
Rugby	2	11.11
Basketball	2	11.11
M aral hon Trial h Ion	2	11.11
Wake skate	1	5.55
Speed skating	1	5.55
Skiing	1	5.55

Among them, 16 patients (88.9%) reported to have been injured while participating in sports, while the two remaining patients (11.1%) were injured as a result of an accidental fall and an accident on the road.

Soccer (football) was the most commonly practiced sport in the sample studied, with 6 out of 18 patients participating.

The median period of follow-up was 65.5 months (IQR 47.5–74.5). Patients were evaluated again to determine the rate of "return to play" and clinical outcome using internationally validated functional scales (PHAT and LEFS). The sample has been analysed considering age, gender, and risk factors in mind, as well as the pathophysiology and anatomy of the injury using Wood’s classification, the time elapsed before surgery, the duration of the rehabilitation protocol, and the eventual return to sports activity, comparing the level of sports performance in the pre- and post-operative periods using the Tegner Activity Score (TAS) (Table 3).

**Table 3:** Frequencies of TAS values measured per-and post-surgery

Tegner activity scale	Pre-op frequency	Post-frequency
9	7	7
8	3	2
7	6	6
4	2	3
17 patients		ΔTAS = 0
1 patient		ΔTAS = 4

VA IN M&M Within the population under study, the "return to play" rate for any sporting activity was 100%. Of the 18 patients that make up the sample, 17 (94%) maintained a level of sports performance comparable to their pre-injury one (Table below). Only one patient was unable to resume his previously practiced sport at pre-injury level but continued practising at recreational level.

The median time elapsed to return to sporting activity was 7 months (IQR 6–11).

Using a yes/no response, each patient was asked to rate their level of satisfaction with the outcome of the surgical treatment of the lesion. 100% of patients deemed the surgical result satisfactory.

The median of the scored obtained by PHAT was 95.0 (IQR 92–99), while the median of the scored obtained by LEFS was 80 (IQR 75–80).

Linear relationship between PHAT and LEFS was established (Rho = 0.58, P-Value 0.01).

The distributions of the collected values for both scores are shown in the following tables (Table 4).

**Table 4:** Frequencies of the values obtained in the functional scores (LEFS e PHAT respectively) reported in the sample

LEFS	Frequency	Percentage
72	1	5.56
73	2	11.11
74	1	5.56
75	1	5.56
78	2	11.11
79	1	5.56
80	10	55.56

PHAT	Frequency	Percentage
83	1	5.56
86	1	5.56
90	1	5.56
92	3	16.67
94	1	5.56
95	4	22.22
97	1	5.56
99	2	11.11
100	4	22.22

The median duration of the rehabilitation protocol within the sample was 4 months (IQR 3–5 months).

Regarding the number of weekly physiotherapy sessions performed, the sample size was split into two categories: the first (intense) includes 9 patients (50%), who performed more than 5 sessions per week (> 5/week). The second (standard) includes as well 9 patients who performed less than 5sessions per (< 5/week). The relationship between the number of weekly physiotherapy sessions and the time to return to sporting activity was investigated (Table 5).

**Table 5:** Duration of rehabilitation protocol, return to sporting activity and functional scores analysed against intensity of physiotherapy, Mann–Whitney test (MW)

Physiotherapy sessions (number)	Standard	Intense	P-value
Duration of rehab protocol (months)	4.0 (4.0–5.0)	3.5 (3.0–5.0)	0.34
Return to sporting activity (months)	7.0(7.0–12.0)	6.0(3,5–7.0)	0.04*
PHAT	95.0 (95.0–99.0)	92 (90.0–99.0)	0.24
LEFS	80.0 (79.0–80.0)	78.0 (74.0–80.0)	0.24

Data expressed as median (IQR)

Patients who adopted an intense physiotherapy regimen returned to sporting activity one month earlier (the difference is significant for *p* value< 0.05). However, a statistically significant result was not found between the two categories and the duration of the rehabilitation protocol.

The correlation between the number of weekly physiotherapy sessions and the results obtained in the functional scores (PHAT and LEFS), also in this case was not statistically significant (*p *value> 0.05).

Taking into consideration the age at surgery and the risk factor of age> 25 years old, younger patients had longer rehabilitation protocol (*p* value< 0.05). The median return to sporting activity was higher in patients aged< 25 years, but the difference in the median times to return to sporting activity in the two age groups was not statistically significant. No significant differences were found regarding the relationship between the two age groups and the scores (Table 6).

**Table 6:** Duration of rehabilitation protocol, return to sporting activity and functional scores analysed against age (older or younger than 25 years old), Mann–Whitney test (MW)

Age at surgery	< 25 years old	≥ 25 years old	P-value
Duration of rehab protocol (months)	4.5 (4.0–6.0)	3.0 (3.0–4.0)	0.02*
Return to sporting activity (months)	7.0 (7.0–12.0)	5.0 (3.3–8.0)	0.07
PHAT	95.0 (92.0–99.0)	95.0 (92.0–99.0)	1.00
LEFS	78,5 (74.0–80.0)	80 (77.5–80.0)	0,24

Data expressed as median (IQR)

Regarding the pathophysiological mechanism of injury patients who have suffered a type 2 injury (overstretch) have a shorter rehabilitation time (*p* value 0.02), but it appears there aren’t any differences in terms of timing of return to sporting activity (Table 7).

**Table 7:** Duration of rehabilitation protocol, return to sporting activity and functional scores analysed against type of Injury (Type 1 – high-speed running and Type 2-overstretch), Mann–Whitney test (MW)

Type of injury	Type 1	Type 2	P-value
Duration of rehab protocol (months)	4.0 (4.0–6.0)	3.0 (3.0–3.5)	0.03
Return to sporting activity (months)	7.0 (6.0–11.0)	7.0 (4.0–9.0)	0,84
PHAT	95.0 (92.0–99.0)	95.0 (92.0–100.0)	1.0
LEFS	80.0 (74.0–80.0)	80.0 (78.0–80.0)	0.81

Data expressed as median (IQR)

## Discussion

Many sports like American football, soccer, rugby, basketball, tennis, and track and field that call for frequent kicking or sprinting are among those that frequently result in hamstring injuries. People who participate in aquatic activities also frequently experience them. These physical activities can result in acute tendon avulsion injuries or lacerations of biomechanically sensitive areas, such as the intratendinous segment or myotendinous junction, due to quick, explosive motions combining hip flexion and ipsilateral knee extension. Patients frequently complain of excruciating throbbing pain over the damaged muscle segment, decreased hip and knee range of motion, and an immediate inability to resume activities [15].

The largest and latest study aiming at understanding epidemiological data on hamstring injuries, conducted over a 13-year period with 263 patients, was released by Irger in 2019. 53% of patients in the study group were male. The majority of patients were between the ages of 45 and 59, with a mean age of 49 ± 13 years. All age groups featured PHAs. Surprisingly, just 4% of the study population, were in the 19–29 age group, while 6% were in the 18 younger age group. This indicates that PHAs primarily affect middle-aged adults and only rarely occur in patients under 30. Older patients may experience severe morbidity after injury, as well as diminished muscle strength, and decreased activity levels [18].

Male patients were significantly disproportionately represented in the age groups of< 18 years, 19–29 years, and 30–44 years, while female patients were older and mostly represented the 45–59 age group. In this study, forceful hip flexion with the ipsilateral knee extended during sports practice was the mechanism of injury that was most frequently reported, followed by falls during daily activities. Sports injuries accounted for 52% of all injuries, according to other research papers, making them the most frequent cause of injuries [19–22].

Male patients were more likely to sustain injuries while participating in sports than female patients, who were predominantly injured while engaging in regular activities Furthermore, patients with sports-related injury were substantially younger and had a significantly lower BMI than patients who were injured during daily activities.

Due to the lack of a uniform classification system, comparisons with other studies regarding the type of injury is restricted. The most frequent type of lesion in the mentioned study was total tendon avulsion with retraction, with involvement of all three tendons (or type 5, according to Wood et al. classification system.). Compared to nonsurgical treatment, surgical intervention yields improved functional outcomes and increased hamstring strength [23]. This finding, however, could be biased by the fact that only surgically treated patients were analysed [2].

In our study, the lesion most commonly found in the sample involved the long head of the biceps femoris (BFlh), which was present in 16 patients (88.9%), followed by lesions of the semitendinosus (ST), which was present in 8 patients (44.4%), and those of the semimembranosus (MS), which was present in 7 patients (38.9%). A total of 13 of the 18 patients examined suffered an injury involving two muscle heads, with 6 of these involving the conjoint tendon, the proximal attachment of the BFlh and ST muscles. Two distinct pathophysiological mechanisms of injury have been identified: type 1 or “high-speed running type” and type 2 or “stretching type”. In accordance with the data in the literature, type 1 lesions affected 13 patients (72.2%) and were predominant.

Among the study population which consisted of 18 patients, 16 of them underwent surgery within six weeks (acute); the two remaining patients underwent surgery between six and twelve weeks (subacute) and more than twelve weeks (chronic). According to Wood’s classification, the most common lesion found in the sample was type 5 (complete avulsion with tendon retraction), which was found in 11 patients (61.1%). Six (33.3%) of the 18 patients in the sample reported having previously suffered hamstring injuries, regardless of severity. One of them claimed to have had calf muscle injuries in the past. Among other risk factors analysed only one patient (5.6%) reported a previous ACL injury.

The relationship between the age factor of 25 and the duration of the rehabilitation protocol, return to sports activity, and functional scores was investigated in our study.

Those who are younger (< 25 years old) have longer rehabilitation protocol duration times when compared to those who are older (*p* value< 0.05). The median return to sport activity was also greater in subjects< 25 years old, but the difference in the median time to return to sport activity between the two age groups was not statistically significant. A possible explanation could be linked to the number of physiotherapy sessions performed by those aged 25 years or more as 5 out of 8 people in this age category had a more intensive physiotherapy regimen. On the other hand, only 4 out of 10 younger patients have received intensive physiotherapy.

Our study additionally revealed no correlation between the two age groups studied and the results obtained in both assessment scores (PHAT and LEFS). A recent study published in the Clinical Journal of Sport Medicine by Ryan P. Coughlin [24] gathered 21 articles for a total of 846 patients with an average age of 41.4 years (range 14–71 y.o.) who underwent surgery following proximal hamstring avulsion injury. The findings revealed a high rate of return to sport after surgery. Overall, the mean time to return to sport was 5.8 months (range 1–36 months).

The study found no significant differences in the rate of return to sport activity when comparing complete versus partial and acute versus chronic avulsions.

The population in our study was younger, with a mean age of 26.4 years (range 14–54 y.o.). Overall median time to return to sport was 7.5 months (range 3–12 months). The return to play rate for any sport participation was 100%, while the return to play rate at pre-injury levels was 94.4%.

There were no relapses in our population sample during the follow-up period. Ten (55.6%) of the 18 patients examined are still active from an athletic point of view, six have given up sporting activity for personal reasons (one of these reported pain at the surgical site, which frequently recurred during sporting activity but never prevented him from continuing), and one patient ended his sporting career 5 years after the operation due to age. At last, only one patient quit his sporting activity because of post-operative complications, as he was no longer able to perform a specific technical gesture inherent in his sport. However, this patient currently practices sports on a recreational basis to this date.

This type of result could be partly explained by the young age of the population under consideration, and it demonstrates surgical treatment of high-grade injuries involving the proximal insertion of the hamstring muscles performed by trained professionals currently represents an effective solution, with a high success rate and a very low rate of complications or recurrences.

Several studies [25, 26] investigating the relationship between operative timing and surgical outcomes have found that surgical repair of a high-grade proximal avulsion is associated with excellent functional outcomes and a high rate of return to sport activity at pre-lesion level, but if the repair is performed after 6 weeks, weakness and difficulty assuming a seated position may occur more frequently, especially if the position is maintained for longer. As a result, early intervention should be preferred. Only two patients underwent surgery after 6 weeks in our study; given the small size of the sample, no statistically significant difference in terms of return to sports activity and functional recovery was observed.

Regarding the nature of the pathophysiological mechanism, several studies have found that type 2 injuries are associated with a poorer prognosis and take longer to recover from [11, 27]. Our study included 5 patients who suffered injury by type 2 mechanism. A correlation between the type of mechanism and the length of the rehabilitation protocol was researched, as well as the return to sports activity and functional scores. Those who have suffered a type 2 injury (overstretch type) have a shorter rehabilitation time, with significant difference. This could be because three of the five patients under investigation received intensive physiotherapy, with more than five sessions per week.

There were no notable differences in terms of time to return to sporting activity. There was no significant difference in the relationship with functional scores.

Numerous studies on the use of post-operative outcome assessment measures following proximal reinsertion of the hamstrings have been published in the last two years. Reza et al. recently published a systematic review [28] (level of evidence 4) of the primary post-operative outcome measures, with the aim of identifying and evaluating the outcome measures used for surgical repair of acute and chronic proximal hamstring avulsions.

Six of the top ten most commonly used outcome measures have been validated: the Lower Extremity Functional Scale (LEFS), the 12-Item Short Form Health Survey (SF-12), the Visual Analogue Scale for Pain (VAS), the Perth Hamstring Assessment Tool (PHAT), the Single Assessment Numeric Evaluation (SANE), and the Tegner Activity Scale (TAS). LEFS has the most items (20) of any of these. The PHAT is the only validated outcome measure for patients with pre- and post-operative proximal hamstring detachments. Blackeney et al. developed this score [29] to assess the functional outcomes of hamstring reattachment surgery based on a prospective series of 74 consecutive surgical repairs in 72 patients, with a mean age of 50,5 years (range 17–74 y.o.).

This study found that there is currently no unambiguous consensus on the best scores for evaluating patients after proximal hamstring avulsion repair; however, it recommended increased efforts to use LEFS and PHAT as patient-reported outcome measures (PROMs) in the evaluation of hamstring injury repair. Another recent study [16] found a strong correlation between PHAT and LEFS, despite the fact that these two PROMs have a weak correlation with objective performance-based functional tests. Based on this evidence, we decided to include these two scores in our study and compare them to the Tegner Activity Score (TAS), which could give us an indication of the true level of sporting activity achieved before and after the intervention. For 17 of 18 patients, the difference in pre- and post-operative TAS scores, expressed as delta TAS (Δ Tas), is equal to zero, demonstrating a high level of return to pre-injury sporting level. The PHAT and LEFS scores additionally provide results that are comparable to the TAS, as both recorded high values for all patients. PHAT and LEFS exhibited good linear link (Rho 0.57, *p* value 0.01). This report highlights how these two functional scores led to the same clinical evidence and can thus be used to assess functional recovery in these patients. Despite the fact that all three scores showed excellent recovery results in our patients, the TAS did not show a significant correlation with PHAT or LEFS. (Rho 0.00 *p* value 1.00 and Rho − 0.15 *p* value 0.54, respectively).

## Conclusions

In our case series, there was a very high rate of return to sport activity following surgical treatment of a proximal avulsion of the hamstring. Almost every patient maintained comparable levels of sport performance to the pre-injury ones: therefore we strongly suggest surgery in active patients who sustained complete hamstrings avulsion.

The high scores measured with two post-operative functional scoring systems, PHAT and LEFS, correlate with the high level of sport performance recovered by our patient. These scores seem to be a reliable system to evaluate the surgical result of hamstring repair surgery in active patients and also top level athletes.

### Limits of the study

The major limits of our study are the small sample size and the retrospective design of the case series.

Although these limits, our case series includes a very homogeneous population for level of sport activity, very high, and age of the patients, almost all young active patients.

These two characteristics of the study are very infrequent in scientific literature about hamstrings avulsion topics.
